# Root growth and function in New Zealand pasture systems: a perspective on research needs, methods, and system integration

**DOI:** 10.3389/fpls.2026.1813059

**Published:** 2026-04-22

**Authors:** Fernando J. Roca Fraga, W. P. T. Wijenayakage, Astrid Volder, Donita L. Cartmill, Katherine N. Tozer, Gercílio A. Almeida, Daniel J. Donaghy, Andrew D. Cartmill

**Affiliations:** 1 School of Agriculture and Environment, Massey University, Palmerston North, New Zealand; 2 Department of Plant Sciences, University of California, Davis, Davis, CA, United States; 3 AgResearch, Ruakura Research Centre, Hamilton, New Zealand; 4 Departamento de Zootecnia, Centro de Ciências Agrárias, Universidade Federal do Espírito Santo, Alegre, ES, Brazil

**Keywords:** belowground, diverse pasture, grazing, pasture persistence, pasture productivity

## Abstract

Understanding root growth and phenology is essential for improving the productivity, resilience, and sustainability of pasture-based systems. However, roots remain one of the most difficult components of plant systems to measure and monitor, particularly in managed, high-turnover pastures, such as those in New Zealand (NZ) dairy systems. As a result, root processes are often underrepresented in both experimental studies and pasture system models. This perspective paper identifies critical, but underdeveloped areas in root research, with particular focus on root phenology. Current studies are limited by insufficient temporal resolution, a lack of species- and cultivar-specific trait data in mixed swards, and weak integration of root dynamics into breeding programmes and farm system models. These constraints limit our ability to link root processes to pasture persistence, nutrient cycling, and climate resilience. To address this gap, we propose that root phenology should be treated as a dynamic functional trait that links plant responses to environmental and management drivers with ecosystem-level outcomes. This framing provides a conceptual foundation for integrating root dynamics into pasture research and modelling, particularly in systems subject to frequent defoliation and environmental variability. We further highlight opportunities arising from rapid advances in sensing technologies, automation, and data analytics, which enable continuous, high-resolution root monitoring systems at multiple scales. However, realising this potential requires integration of complementary measurement approaches and alignment with system-level research questions. In this context, NZ provides a unique platform for developing scalable, pasture-based root monitoring framework that integrates science, management and policy. We argue for a coordinated effort that bridges fundamental root biology with applied pasture management, supported by long-term datasets, methodological integration, and engagement with end users. Embedding root traits and phenological dynamics into the next generation of pasture models and decision-support tools will be critical for improving system performance and environmental outcomes. This perspective aims to stimulate a shift towards more integrated, temporally explicit approaches for studying root systems in pasture environments, with relevance to grazing system beyond NZ and across temperate regions.

## 1 Introduction

Fine roots serve as a critical interface between plants and soil, underpinning water and nutrient acquisition, carbon (C) inputs, and microbial activity in pasture systems (Dunbabin et al., 2002; Klumpp et al., 2009; McNally et al., 2015). Despite their ecological and agronomic importance, root systems remain among the least understood components of pasture agroecosystems, particularly in terms of their growth dynamics, spatial organisation, turnover, and temporal development. This is especially true in managed pasture systems, where repeated grazing, seasonal variability, and soil heterogeneity interact to shape belowground responses over relatively short time scales. Pasture species vary widely in their root traits, including total and fine root biomass, rooting depth, vertical distribution, turnover rates, and morphological characteristics (Reid et al., 2015; Yang et al., 2017), yet these ‘dimensions’ of plant function are rarely monitored with the same temporal resolution or interpretive depth as aboveground traits.

Plant functional traits provide a mechanistic basis for linking pasture management, environmental variation, and ecosystem processes. Traits such as specific root length, root diameter, tissue density, root nitrogen (N) concentration, specific leaf area, and shoot and root C:N ratio have been associated with variations in plant productivity, persistence, and forage quality (Craine et al., 2001; Marshall et al., 2016). However, belowground traits remain underrepresented in pasture research, despite their importance for nutrient capture, drought tolerance, regrowth capacity, soil structure, and long-term system resilience. This imbalance reflects both methodological challenges and a broader bias within pastoral research and extension systems toward aboveground indicators that are easier to measure and interpret.

Pasture management strongly influences root growth and function. Stocking rate, stocking density, grazing intensity, grazing interval, fertilizer use, irrigation, and pasture species composition all affect root production, allocation, turnover, and regrowth and recovery dynamics (Greenwood and Hutchinson, 1998; Greenwood and McKenzie, 2001; da Silva et al., 2019). In grazed systems, defoliation represents a recurrent disturbance that alters photosynthetic capacity and shifts plant C allocation priorities. Root production often slows after defoliation (Volder et al., 2007, 2015), reflecting reduced assimilates supply to belowground tissues, while subsequent recovery (regrowth) depends on carbohydrate reserves, shoot regrowth, species identity, and prevailing environmental conditions (Donaghy and Fulkerson, 1998; Tozer et al., 2017; Moot et al., 2021). Abiotic factors, including soil compaction, temperature, moisture, nutrient status, and seasonal variability, further interact with management practices to shape root growth trajectories and recovery potential (Greenwood and McKenzie, 2001; Dodd and Mackay, 2011). Managed pastures systems therefore provide a valuable model for examining root functional traits and temporal dynamics under repeated disturbance, with insights extending beyond agricultural systems.

Roots also play a central role in delivering ecosystem services (ES) in pasture systems. Through rhizodeposition, turnover, and interaction with the soil matrix, roots promote aggregate formation, influence porosity, enhance infiltration, and contribute to erosion control and water retention, particularly in intensively grazed or topographically variable landscapes (Kuzyakov et al., 1999; Rillig et al., 2015). Fine root turnover and associated rhizosphere processes also contribute to soil organic matter (SOM) formation, including C inputs to subsoil horizons, where turnover and decomposition dynamics may differ from surface soils (Lal, 2004; Kell, 2012). In addition, root systems regulate nutrient retention and cycling by intercepting mineral nutrients, redistributing water, and reducing the risk of nutrient loss to surface and groundwater.

Roots also exert strong control over soil biological processes. Root exudates influence microbial composition and activity, shaping nutrient mineralisation, soil aggregation, and plant-soil feedbacks that regulate pasture performance. In mixed swards, including those containing legumes, root-associated microbial processes contribute to biological N fixation and broader soil biodiversity, reinforcing system resilience under environmental variability (Bardgett et al., 2014; Hu et al., 2021). Root systems also influence microbial and fungal community structure through associations with mycorrhizal fungi and other beneficial microorganisms that affect plant nutrient uptake, pathogen resistance, and tolerance to climatic stress. Recent work has highlighted links between rooting depth gradients and microbiome composition, suggesting that root distribution through the soil profile has implications not only for resource capture, but also for belowground biological function (Murray et al., 2025). Despite the importance of these processes, most ES assessments remain predominantly aboveground in focus and/or rely on simplified models of belowground dynamics.

This gap is particularly important in NZ, where pasture-based livestock systems are strongly dependent on soil-plant interactions and where environmental performance is increasingly scrutinised in relation to water quality, nutrient losses, greenhouse gas (GHG) emissions, and long-term soil health. In this context, improved understanding of root dynamics has relevance not only for pasture persistence and productivity, but also for system-level outcomes including nutrient use efficiency, drought resilience, C storage, and freshwater protection.

A key, yet underdeveloped, dimension of belowground function is root phenology. While many pasture studies have quantified root biomass, root length, or rooting depth, less attention has been given to the timing of root initiation, elongation, senescence, turnover, and post-defoliation recovery. These temporal dynamics are critical because they determine when roots are actively acquiring resources, contributing C to the soil, and recovering following disturbance. In pastoral systems, root phenology is shaped by grazing and cutting events, seasonal climatic variability, and management inputs such as fertilisation and irrigation. Root regrowth may be delayed following grazing (Volder et al., 2007, 2015), particularly under intensive management or drought, affecting nutrient uptake, soil stability, and subsequent pasture recovery (Skinner and Comas, 2010; Teague and Kreuter, 2020). The magnitude of these effects depends on grazing severity, with more frequent and closer defoliation often retarding root recovery (Donaghy and Fulkerson, 1998).

Unlike aboveground phenology, root phenology is difficult to observe directly, yet it governs key processes in plant resource allocation and belowground C dynamics. Repeated minirhizotron (MR) imaging, isotopic tracing, and temporally resolved root coring can reveal the dynamics of root production, growth, turnover, and senescence, but these approaches remain technically demanding and are rarely deployed at sufficient temporal resolution to capture short-lived responses or post-defoliation recovery dynamics. As a result, dynamic root processes remain poorly represented in empirical datasets and modelling frameworks. For example, current pasture system models, such as OverseerFM and APSIM-NZ, typically simplify belowground processes and underrepresent dynamic root traits, limiting the ability to link root activity to productivity, nutrient cycling, and soil C storage. This limitation is particularly important in highly managed systems where temporal mismatches between root activity, nutrient availability, and grazing events influence nitrate (NO_3_^-^) leaching, pasture resilience, and seasonal resource-use efficiency.

Methodological constraints have contributed to this knowledge gap and continues to limit our capacity to study root systems at appropriate temporal and spatial scales (**Table 1**). The belowground environment is complex and heterogeneous, difficult to access, and costly to characterise at appropriate spatial and temporal scales. Historically, pasture studies have prioritised short-term aboveground measurements, while root assessments have often been infrequent, destructive, and insufficiently integrated with soil, microbial, and system-level data. However, advances in sensing technologies, automation, image analysis, isotopic approaches, and data integration now provide new opportunities to study root systems with greater temporal resolution and mechanistic insight.

**Table 1:** Summary of key constraints limiting pasture root system research.

Problem/constraint	Explanation
Limited temporal and spatial resolution	- Most current root phenology studies are constrained by infrequent sampling intervals and spatially coarse observations. - This limits our understanding of fine-scale root dynamics, particularly in response to short-term climatic variability, grazing events, or shifts in soil water availability. - Improved temporal resolution is especially critical in systems where root production and mortality can be highly episodic and asynchronous with shoot growth.
Underrepresentation of diverse species and systems	- Root studies have disproportionately focused on a limited number of species, particularly temperate forest trees and model organisms like *Arabidopsis thaliana* (L.) Heynhold and *Medicago truncatula* Gaertn., while agriculturally and ecologically significant systems such as perennial pasture grasses remain relatively underrepresented. - This is especially true for managed grasslands, where root dynamics can vary widely among species and functional groups due to differing growth strategies, defoliation responses, and symbiotic associations.
Disconnect between root traits and ecosystem functions	- Root biomass and turnover have been broadly studied, but the relationship between root phenology and ecosystem services including carbon (C) sequestration, nutrient cycling, and water use efficiency, remains poorly quantified. - There is a need for trait-based approaches that link observable root characteristics (e.g. lifespan, tissue density, mycorrhizal status) with ecosystem processes and long-term sustainability goals.
Limited integration of root and shoot phenology	- Root and shoot phenology are often decoupled in both measurement and modelling frameworks, limiting holistic understanding of plant functioning. - Many models of plant productivity or C cycling rely on shoot-based inputs and do not adequately capture belowground dynamics. - New integrative frameworks are needed to synchronize root and shoot phenology data for system-level interpretation.
Inadequate methodological validation	- Existing methods for measuring root dynamics vary in precision, scale, and repeatability. - Few studies compare multiple methods under identical field conditions, leading to uncertainty in root growth estimates and bias in data interpretation. - For example, comparisons of minirhizotron imaging with root coring, or isotope labelling with biomass harvest, are rarely published. - Benchmarking studies are critical for selecting appropriate methods under different system constraints.

In this perspective, we argue that root phenology should be treated not simply as a descriptive aspect of root growth, but as a dynamic functional trait linking plant response to environmental and management drivers with their effects on ecosystem processes. We use this perspective to reframe the role of roots in NZ pasture systems, identify key methodological and conceptual gaps, and outline pathways for integrating root dynamics into pasture science, modelling, and management. Our aim is not to provide an exhaustive review, but to stimulate discussion around the incorporation of root phenology and function into the next generation of pasture research and systems integration.

## 2 Root phenology as a dynamic functional trait: a conceptual framework

Root phenology is widely invoked in plant and ecosystem studies, yet it is rarely defined or operationalised in a way that allows it to function as a comparative or predictive trait across systems. In pastoral contexts, the term is often used broadly to describe the timing of root initiation, elongation, senescence, and turnover, particularly in response to seasonal conditions and grazing events (Moot et al., 2021; Cui et al., 2024; Bonato Asato et al., 2025). However, for root phenology to contribute meaningfully to pasture science, modelling, and management, it must be conceptualised not simply as a descriptive set of processes, but as a dynamic functional trait that integrates plant responses to environmental and management drivers with downstream effects on ecosystem function.

Within this perspective, root phenology is defined as the temporal dynamics of root initiation, elongation, lifespan, turnover, and post-defoliation recovery in response to environmental and management drivers. These components can be quantified through metrics such as root length production rates, lifespan distributions derived from MR observations, turnover rates, and temporal variation in root length density. Framing root phenology in this way enables comparison across systems and provides a basis for linking root dynamics to measurable outcomes in plant performance and ecosystem processes.

In trait-based ecology, functional traits are commonly classified as either response traits, which determine how plants respond to environmental conditions, or effect traits, which determine how plants influence ecosystem processes (Garnier and Navas, 2012; Díaz et al., 2013; Muler et al., 2018). While this distinction has been widely applied to aboveground traits, its application to belowground systems (particularly temporal dynamics) remain underdeveloped. Root phenology provides a natural bridge between these domains. The timing and rate of root initiation, elongation, and regrowth reflect plant responses to soil moisture, temperature, nutrient availability, and defoliation. At the same time, root lifespan, turnover, and exudation patterns exert strong controls over soil C inputs, nutrient cycling, microbial activity, and water dynamics. In this sense, root phenology cannot be adequately described as either a ‘response’ or an ‘effect’ trait alone, rather, it represents a temporal functional axis that links these two domains.

This perspective aligns with, and also extends, existing trait-based frameworks such as the root economics spectrum (RES) (Suding et al., 2008; Freschet et al., 2010; Reich, 2014; Faucon et al., 2017; Kong et al., 2019). The RES describes trade-offs between resource acquisition and conservation strategies through traits such as specific root length (SRL), tissue density, and root diameter. However, it does not explicitly account for the timing, duration, and synchronisation of root activity. Root phenology addresses this gap by describing when roots are produced, active, and lost, thereby influencing the temporal coupling between plant demand and soil resource availability. Within this context, root phenology can be interpreted as a ‘temporal regulator’ of trait expression along the RES, influencing when acquisitive or conservative strategies are deployed under variable environmental and management conditions.

This temporal dimension of root function is particularly important in managed pasture systems, where repeated defoliation events impose strong and recurrent disturbances to plant C balance. Grazing or cutting events reduce photosynthetic capacity and alter C allocation priorities, often leading to short-term reductions in root growth and, in some cases, increased root mortality. Subsequent recovery depends on the availability of stored carbohydrates, environmental conditions, and species-specific strategies governing regrowth. These dynamics give rise to defoliation-driven C allocation trade-offs, in which plants must balance investment in shoot regrowth to restore photosynthetic capacity against the maintenance and regeneration of root systems required for resource acquisition and persistence. Root phenology plays a central role in mediating these trade-offs. Delays in root regrowth following defoliation can reduce nutrient uptake during critical recovery periods, while rapid root turnover may enhance soil C inputs but reduce long-term resource capture efficiency. Conversely, deeper or more persistent root systems may support drought resilience and nutrient retention but require sustained C investment. Variation in these temporal dynamics therefore has direct implications for nutrient uptake efficiency, soil C inputs, pasture recovery, and longer-term system resilience, linking root phenology explicitly to both plant performance and ecosystem function.

**Figure 1:**
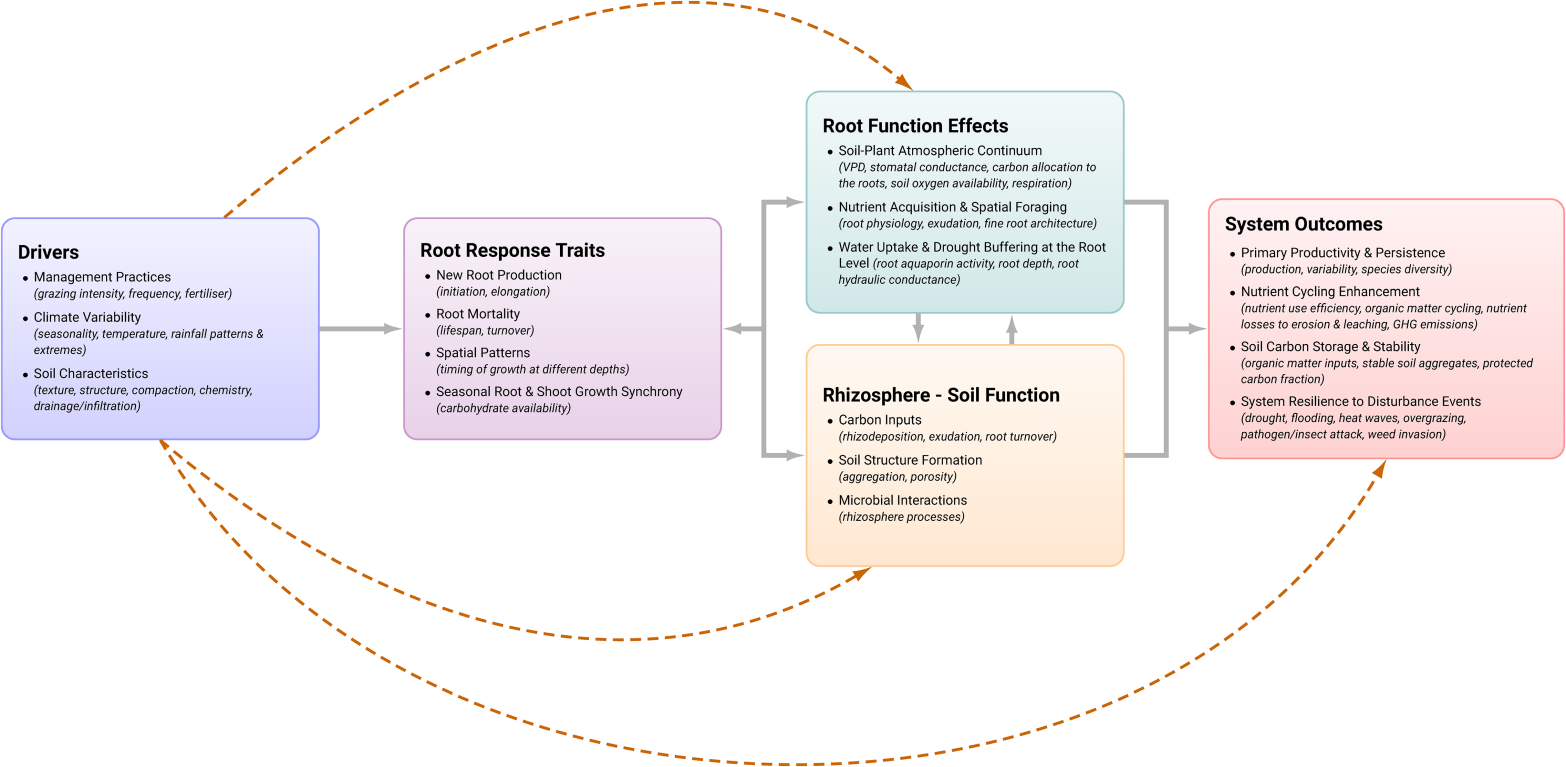
Conceptual framework positioning root phenology as a dynamic functional trait linking plant response to environmental and management drivers with ecosystem outcomes. Solid arrows indicate primary directional pathways across the framework, while dashed arrows represent additional linkages.

To formalise these linkages, we propose a conceptual framework (**Figure 1**) in which root phenology is positioned as a dynamic interface connecting management, plant physiology, and ecosystem processes. In this framework, external drivers, including grazing intensity and timing, climatic variability, soil physical and chemical conditions, and management inputs such as fertilisation and irrigation, shape root phenological responses, including initiation, elongation rates, lifespan, turnover, and post-defoliation recovery dynamics. These responses are mediated through shifts in C allocation between aboveground and belowground compartments, reflecting both immediate physiological constraints and longer-term adaptive strategies.

Root phenological responses influence a suite of effect pathways, including nutrient uptake dynamics, soil C inputs through root turnover and rhizodeposition, soil structure and aggregation, and interactions with microbial communities. These processes collectively determine key system-level outcomes, including pasture productivity, persistence, nutrient use efficiency, water regulation, and resilience to climatic stress. Importantly, these relationships are not unidirectional. Feedbacks between root activity, soil conditions, and plant performance create dynamic, non-linear interactions that evolve over time and across management regimes.

Positioning root phenology within this framework has several implications: i) It highlights the need to move beyond static measurements of root biomass and distribution toward temporally explicit metrics that capture rates of growth, turnover, and recovery; ii) It emphasises the importance of aligning measurement approaches with key management and environmental transitions, particularly in highly dynamic systems such as grazed pastures; iii) It provides a basis for integrating root processes into pasture models and decision-support tools, where temporal mismatches between root activity and resource availability can have significant implications for nutrient losses, productivity, and environmental outcomes. In the context of NZ pastoral systems, this framework is particularly relevant. The combination of intensive grazing, climatic variability, and increasing regulatory pressure on nutrient losses creates a strong need to better understand how root dynamics influence system performance. In practice, modelling platforms such as OverseerFM and APSIM rely on simplified representations of root growth and turnover, limiting their ability to capture the effects of grazing, defoliation timing, and species-specific root phenology on nutrient fluxes and pasture resilience. Incorporating root phenology as a dynamic trait provides a pathway to improve representation of belowground processes, particularly in relation to N cycling, C dynamics, and seasonal variability in resource uptake.

By framing root phenology as a dynamic functional trait that integrates response-and-effect pathways, this perspective provides a conceptual foundation for advancing root research in pasture systems. This framework complements existing trait-based approaches by introducing a temporal dimension that is essential for understanding and managing ‘disturbance-driven’ agroecosystems.

## 3 Root monitoring in pasture systems: status and limitations

Much of what is known about plant phenology is based on aboveground measurements (e.g. leaf emergence, flowering, and senescence) (Radville et al., 2016a; Weigelt et al., 2021), however, despite growing recognition of the importance of root systems in plant productivity, resource cycling, and ecosystem resilience, root phenology remains one of the least understood aspects of plant biology (Wardle et al., 2004; McCormack et al., 2017; Freschet et al., 2021). This imbalance limits our ability to predict belowground responses to environmental variability, seasonal dynamics, climate stress, and pasture management interventions.

Within the conceptual framework outlined in Section 2, this limitation reflects a lack of data to resolve key components of root phenology, including initiation, elongation, lifespan, turnover, and post-defoliation recovery. As a result, the temporal dynamics linking plant responses to ecosystem processes remain poorly constrained, particularly in highly managed pasture systems where disturbance is frequent and system responses are rapid. For example, root growth often exhibits seasonal patterns that do not directly align with aboveground phenology (Radville et al., 2016b; McCormack et al., 2017), further complicating interpretation of plant responses based solely on shoot-based measurements. In grazed systems, defoliation represents a key driver of root phenology, yet its effects remain insufficiently resolved. While numerous studies have demonstrated that defoliation events reduce root biomass (Schuster, 1964; Greenwood and Hutchinson, 1998; McNaughton et al., 1998; Mapfumo et al., 2002), relatively few studies have ‘disentangled’ short-term responses, such as reduced C allocation to roots, from long-term adaptive responses involving recovery, compensation, and shifts in allocation patterns. This limits our ability to quantify defoliation-driven C allocation trade-offs, which are central to the conceptual framework proposed in this perspective manuscript. In addition, there is limited information on how root phenological dynamics vary across functional groups (i.e. grass, broadleaf, and legume), species, and cultivars, particularly in diverse pasture swards typical of NZ systems. Difference between grasses, legumes, and forbs in rooting depth, turnover rates, and recovery strategies are likely to influence system-level outcomes, yet these variations remain poorly characterised. Trait-based approaches that integrate root structure with temporal dynamics are underdeveloped in pasture systems compared with forest or cropping systems. Root turnover and exudation represent key pathways for SOM inputs (Kell, 2012; McNally et al., 2015), yet few studies in pastoral systems have directly quantified these processes or linked them to temporal variation in root activity. This represents a critical knowledge gap, as variation in root lifespan and turnover influences C inputs to soil, nutrient cycling, and microbial activity, with implications for long-term soil function and system resilience. These limitations are further reflected in current modelling approaches. For example, in practice, platforms such as OverseerFM and APSIM rely on simplified representations of root growth and turnover, limiting their ability to capture the effects of grazing, defoliation timing, and species-specific root phenology on nutrient fluxes, C dynamics, and pasture resilience. The lack of temporally explicit root data constrains model parameterisation and reduces the capacity to simulate key processes such as N uptake, NO_3_^-^ leaching, and seasonal variability in resource use. Taken together, these knowledge gaps highlight a disconnect between the recognised importance of root systems and the availability of data required to characterise their temporal dynamics. Addressing this gap requires improved measurement approaches capable of resolving root phenology at appropriate spatial and temporal scales, as well as integration of these data with aboveground, soil, and microbial processes. These challenges underpin the need for methodological integration, which is explored in the following section.

## 4 Measuring root growth in pasture systems: synergies and integration of methods for root characterization

A wide range of techniques are available for studying root growth and phenology in pasture systems, each offering distinct trade-offs in terms of cost, accuracy, labour requirements, and spatial-temporal resolution (**Table 2**). Our aim is not to provide an exhaustive overview of methodological approaches, but rather to highlight how method must be carefully aligned with study objectives, sampling scale, site conditions, available expertise, and logistical constraints. Considerations such as topography, soil texture, compaction, and moisture levels influence both feasibility and data quality. Increasing the frequency of sampling can improve detection of short-term root responses, such as post-grazing regrowth, but also increases labour and cost.

**Table 2:** Comparative overview and synthesis of root characterisation methods.

Method	Type	Purpose	Strength	Limitation	Best practice	Synergy	Integration	Key references
Soil coring	Destructive	Estimate root biomass, depth distribution	Simple, accessible; quantitative Can be depth-stratified - Enables biomass quantification	Destructive; low temporal resolution; labour intensive	Stratify by depth; replicate across space/time; pair with moisture/temp data	Biomass and root length density by depth	Combine with isotopes, DNA, or Ground Penetrating Radar for added insight	Böhm (1979); Neill (1992); Wasson et al. (2014)
Monolith excavation	Destructive	Detailed root architecture and spatial mapping	High-resolution root structural data Captures intact root architecture - Whole-system perspective	Highly labour intensive; destructive; hard to scale High costs (labour, transport, and processing) - Soil-type sensitive	Use when whole-plant structure needed; mechanised aid for large monoliths	Architecture, depth, root-soil interaction	Use with image-based analysis or mycorrhizal quantification	Böhm (1979); Nelson and Allmaras (1969); Buman et al. (1994); Teramoto et al. (2019); Allaire and Bochove (2006); Henry et al. (2011)
Minirhizotron	Non-destructive	Monitor root growth and turnover over time	Non-destructive; repeatable time-series data	Costly; requires image analysis Limited field of view - Expensive setup - Needs calibration	Standardised install; frequent imaging; calibrate with destructive sampling Monitoring seasonal root growth and turnover dynamics in situ	Root growth dynamics, turnover, species-specific root timing	Ground-truth with destructive sampling	Samson and Sinclair (1994); Majdi (1996); Johnson et al. (2001); Joslin and Wolfe (1999)
Carbon isotope tracing (δ¹³C)	Biochemical	Assess root function, turnover, C dynamics/allocation	Integrates physiological function; links above and belowground	Sensitive to species, depth, OM content; lab-based - High variability in pasture systems - Root-source signal may be obscured	Stratify by depth; avoid mixed age/condition roots; calibrate against known standards Studying C dynamics and linking root function to environmental conditions	Water use efficiency, photosynthetic pathway tracking	Combine with root washing and stratification	Brüggemann et al. (2011); Alexandre et al. (2016); Rewald et al. (2012)
Magnetic resonance imaging (MRI)	Non-destructive	- High spatial resolution - Enables time-series tracking - Visualizes root architecture	Expensive and technically complex - Often limited to pot-based or controlled settings	Soil properties may interfere with signal -Limited by high organic matter, low water, etc. Limited spatial resolution for fine root detection	High-resolution root imaging in controlled experiments or calibration studies	Fine roots detection, mapping root zones	Ground-truth with destructive sampling	Pflugfelder et al. (2017); Van Dusschoten et al. (2016); Rogers and Bottomley (1987); Pohlmeier et al. (2008)
Ground penetrating radar (GPR)	Remote sensing	Map coarse root systems non-destructively	Non-invasive; broad coverage - Non-invasive spatial mapping of coarse roots	Lower resolution in moist, clay-rich soils; requires calibration - Limited resolution for fine roots - Soil moisture/clay content affect penetration	Combine with coring; optimise antenna for soil type; ground-truth models Mapping root system extent when calibrated with ground-truthing	Coarse root detection, mapping root zones	Ground-truth with coring or monoliths	Liu et al. (2018); Guo et al. (2013); Wolfe et al. (2021); Liu et al. (2016)
Electrical resistivity tomography (ERT)	Remote sensing	Detect root zones via soil resistance - Detects variation in root-zone conductivity - Suitable for larger field areas	Spatially extensive root zone mapping - Requires site-specific calibration - Sensitive to soil heterogeneity	Influenced by soil moisture, salts; requires expertise	Use with other data sources (GPR, cores); site-specific calibration Exploratory mapping of root zones or root water uptake areas	Coarse root detection, mapping root zones	Ground-truth with coring or monoliths	Srayeddin and Doussan (2009); Garré et al. (2011); Vanella et al. (2018); Rao et al. (2020); Rossi et al. (2011)
Combined/Integrated Methods	Combined	Cross-validate, enhance resolution and reliability	Multi-dimensional insight; system-level understanding	Requires coordination; cost and complexity increase	Combine destructive and non-destructive tools; integrate environmental sensors; use AI^1^/ML^2^ where feasible	Links root structure, biomass, and dynamics across scales	Combined methods for robust validation and system-level insight	Rewald et al. (2012); Paez-Garcia et al. (2015); Maeght et al. (2013); Cabal et al. (2021); (Rewald et al. (2012); Nair et al. (2023);
Artificial intelligence (AI) models & machine learning (ML) algorithms	Analytical	Cross-validates, enhances resolution and reliability	Can guide sampling, pattern detection, and potentially improves with use	Dependent on quality of input data - Still under development	To optimise data use, reduce labour, and extrapolate trends	Pattern detection, real-time monitoring	Augments all methods; boosts interpretation efficiency	Han et al. (2021); Hiraiwa et al. (2019); Weihs et al. (2024); Handy et al. (2024)

^1^Artifical intelligence (AI); ^2^ Machine learning (ML).

In highly dynamic pasture systems, seasonal or annual sampling is insufficient to capture root turnover rates as a significant fraction of fine roots may appear and die between sampling dates. Targeted sampling around key phenological or management transitions, including grazing events, fertilizer application, droughts, or seasonal changes, can provide more meaningful insight into temporal root dynamics. There is therefore a strong need to integrate available methods and better balance precision, scalability, and ecological relevance. A diverse set of complementary approaches exist for monitoring root systems, and each method, whether destructive or non-destructive, captures different facets of root system dynamics (**Table 2**). No single technique is ‘universally optimal’, and each offers strengths which are best suited to specific research questions. Destructive methods such as soil coring, are the ‘gold standard’ for quantifying standing root biomass, root length, and trait diversity. However, they are labour intensive, limited in spatial representativeness, and provide only a snapshot of root systems at a single point in time, making them poorly suited to capturing temporal dynamics. Non-destructive tools, including MR systems and ground penetrating radar (GPR) enable repeated observations of root growth and turnover, improving temporal resolution. However, MR installation can alter soil structure, and root proliferation at the tube interface may not reflect true field conditions (Joslin and Wolfe, 1999; Withington et al., 2003). In addition, image processing remains time-intensive, although advances in automated analysis are reducing this limitation. Ground penetrating radar provides broader spatial coverage but loses resolution with depth and is less effective at detecting fine roots. Isotopic and molecular approaches provide additional functional and taxonomic insights, particularly in complex multispecies systems. Stable isotope tracing can be used to investigate C allocation, water uptake, and nutrient dynamics, while molecular techniques allow characterisation of root-associated microbial communities. These approaches are valuable for linking root phenology to ecosystem processes, but are often constrained by cost, technical complexity, and challenges in interpretation.

Given these limitations, an integrated, multi-method approach is required to adequately characterise root systems in pasture environments. Combining destructive and non-destructive approaches enables calibration, validation, and improved interpretation across spatial and temporal scales. For example, soil coring can be used to ground-truth MR or GPR observations, while stratified sampling by depth, phenological stage, and site conditions improves contextual understanding. Pairing remote sensing approaches with targeted sampling can enhance spatial extrapolation of root traits and dynamics, and combining soil cores with MR, GPR, or electrical resistivity tomography (ERT) allows multi-scale observation of root structure, turnover, and distribution.

Integration across biological and environmental domains further strengthens interpretation. For example, linking root measurements with aboveground biomass measurements enables analysis of root–shoot allocation to new growth, regrowth potential, and grazing recovery. Combining root biomass assessments with soil organic C (SOC), microbial activity, and soil respiration offers insight into the role of root systems in C cycling and sequestration under pasture management regimes (Paustian et al., 2000; Kell, 2012). Similarly, integrating root samples with microbial DNA analysis would allow for exploration of root–microbiome interactions, including N-fixing bacteria in legume-based swards or mycorrhizal associations that support nutrient acquisition and drought resilience (Pantigoso et al., 2022; Ragland et al., 2024; Yusuf et al., 2025).When combined with environmental sensors measuring soil temperature and moisture at multiple depths, MR imaging provides insight into root responses to fluctuating environmental conditions. Stable isotope tracing (e.g. δ¹³C, δ^2^H, or δ^15^N), when used alongside MR data and biomass sampling can reveal spatial and temporal patterns of water uptake (Beyer et al., 2016, 2018; Penna et al., 2020) and C partitioning to the root system (Williams et al., 1991; Boutton et al., 1999). These integrated datasets are critical for linking root phenology to system-level processes and for improving model parameterisation.

While integrated approaches improve resolution and interpretation, they also introduce complexity. Many methods require specialised expertise, are time-intensive, and involve substantial upfront costs. Advances in machine learning (ML) and AI offer compelling opportunities to improve scalability and efficiency. Automated image recognition, including convolutional neural networks, can accelerate root identification and analysis from MR and imaging datasets, while ML approaches can assist in pattern detection, sampling design, and data integration (Nadezhdina and Čermák, 2003; Van Dusschoten et al., 2016). These tools have the potential to reduce labour requirements and improve reproducibility, although they require careful validation.

Overall, no single method is sufficient to characterise root systems across space and time. Integrated, multi-method strategies are therefore essential for resolving root phenology, linking root dynamics to ecosystem processes, and supporting the development of predictive models of belowground function in pasture systems.

## 5 Strategic pathways for advancing root research

**Figure 2:**
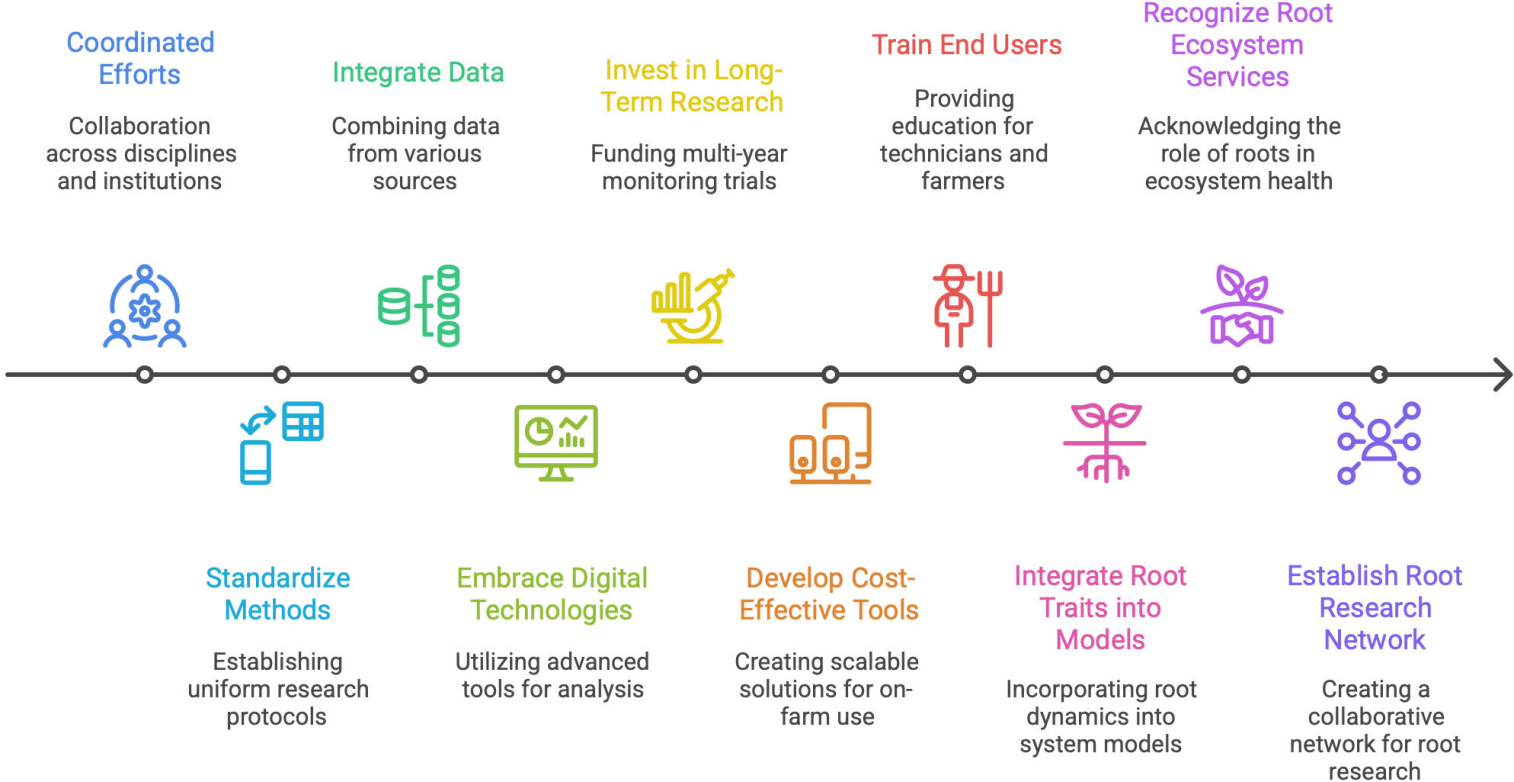
Strategic pathway for advancing root research in New Zealand.

Advancing root research in NZ pasture systems will require coordinated efforts across disciplines, institutions, and sectors. While new technologies and data tools hold promise, strategic prioritisation and investment are essential to move from isolated studies to system-wide understanding. Progress will depend on improving the capacity to quantify root phenology as a dynamic functional trait to integrate these processes into pasture management, modelling, and policy frameworks. To achieve this, coordinated efforts are required to standardise methods, integrate data across scales, embrace digital technologies, and invest in long-term system-based research. These strategic pathways are not mutually exclusive, rather, they are mutually reinforcing. Their implementation will accelerate our understanding of root dynamics, improve decision-making in pasture management, and help support outcomes related to climate resilience, soil health, and sustainable intensification in grazing systems. Rather than treating roots as passive nutrient gatherers or structural anchors, they should be framed as dynamic interfaces linking plant physiology, soil processes, and microbial communities. This system perspective enables roots to be evaluated not only in terms of biomass or morphology, but in terms of their contribution to soil health, water dynamics, nutrient cycling, and pasture resilience under climate stress. Embedding root traits and phenological dynamics into whole-system models, such as pasture growth or nutrient budgeting tools, can help bridge the gap between plant physiology and farm-scale decision-making (Lynch, 2019). We therefore propose a strategic pathway to advance root research in NZ (**Figure 2**).

A critical limitation in current root research is the lack of long-term datasets capturing root dynamics across seasons, grazing cycles, climatic variability, and management regimes. Most existing studies are short-term, limiting the ability to identify thresholds, lag effects, and compensatory responses in root systems and root-shoot allocation. Investment in multi-year monitoring trials that integrate aboveground production, belowground dynamics, soil properties, and environmental variables would provide essential insight into the temporal processes highlighted in this perspective, including defoliation-driven allocation trade-offs and feedbacks between root activity and soil processes. These datasets are also necessary for validating remote sensing approaches and improving model representation of root dynamics. These efforts should encompass a range of systems, including ryegrass-clover pastures, dryland systems, and native or semi-natural grasslands, where the drivers and functional importance of root phenology differ. Longitudinal approaches are particularly valuable for quantifying root turnover, decay rates, and legacy effects associated with pasture renovation, drought, or grazing exclusion.

Improving the scalability and accessibility of root measurement is another major priority. Many current approaches remain labour intensive and are impractical for routine field application. Future research should prioritise adaptable, cost-effective tools capable of deployment at the paddock scale, including lower-cost imaging platforms, mobile MR units, or AI-assisted soil core analysis. However, technological innovation must be accompanied by usability testing under ‘real farm’ conditions, along with training programmes for a broader range of users, including technicians, consultants, and farmers. Open-source databases and ‘analytical pipelines’ could further improve consistency, reduce duplication of effort, and accelerate knowledge transfer.

The integration of diverse root datasets (e.g. imaging, coring, remote sensing, isotope analysis, etc.) offers significant opportunities for advancing understanding, but also introduces challenges related to data volume, variability, and standardisation. Machine learning approaches, particularly when combined with climate, soil, and management metadata, can improve pattern detection, optimise sampling strategies, and enhance performance (Atkinson et al., 2019; Weihs et al., 2024). Interdisciplinary collaboration between plant ecologists, soil physicists, computer scientists, and modellers will be essential to unlock these opportunities. New Zealand is well positioned to lead in this space, given its existing strengths in pasture research, environmental monitoring, and modelling frameworks. Policy frameworks must also evolve to better recognise the role of roots in delivering ES, including soil C storage, erosion control, nutrient retention, and drought resilience. Current indicators and incentive systems often overlook root-related processes, despite their importance in forming stable soil C pools (Rasse et al., 2005; Sokol et al., 2019). The development of root-informed indicators and guidelines for NZ pasture systems would improve alignment between farm managers, environmental outcomes, and regulatory objectives. This could include targeted investment in root phenotyping trials, soil C calibration efforts, and the incorporation of root metrics into pasture health assessments.

A further constraint is the limited number of researchers trained in both plant and soil root dynamics. Addressing this gap will require investment in technical training, root phenotyping expertise, and standardisation of field and laboratory protocols. National collaboration, potentially through a dedicated root research network aligned with existing organisation, could support shared infrastructure, long-term experiments, and methodological consistency across institutions. The development of root trait databases and benchmark reference sites across key agroecological zones in NZ would further support model parameterisation and cross-site comparison. Integrating these efforts into existing long-term pasture experiments would ensure that root dynamics are captured alongside aboveground and environmental measurements. This also supports a shift from descriptive studies towards systems-based approaches that integrate belowground processes into pasture models, life-cycle assessments, farm economic models, and soil-plant-animal feedback frameworks.

Ensuring that root research translates into practical outcomes requires strong engagement with end users. Participatory approaches, including farmer involvement in experimental design, scenario testing, and decision-support tool development, can help align research priorities with on-farm needs. Linking root traits and phenological dynamics to measurable outcomes, such as pasture recovery, nutrient losses, and drought resilience, will be essential for driving adoption and demonstrating value. However, despite these opportunities, challenges remain, particularly related to cost, technical complexity, and scalability. Addressing these constraints will require sustained investment and coordinated effort. However, by aligning methodological innovation, data integration, and system-level thinking, there is substantial potential to advance root research and improve the integration of belowground processes into pasture systems, modelling frameworks, and environmental management.

## 6 Conclusion

New Zealand’s pastoral systems are globally distinctive, relying heavily on year-round grazing of perennial swards rather than feedlot or forage crop-based feeding systems (Clark et al., 2007). This reliance on soil-plant interactions places root systems at the centre of productivity, resilience, and sustainability. However, roots remain the most under-characterised component of these systems. Improved root monitoring offers significant potential to enhance understanding of pasture persistence, water and nutrient use efficiency, and soil C storage, all of which are increasingly important under climate change and tightening environmental regulation (de Klein et al., 2022). Quantifying root growth and phenology in pasture systems requires careful consideration of trade-offs between spatial resolution, temporal sensitivity, destructive versus non-destructive techniques, and resource constraints. Traditional approaches, such as soil coring and monolith extraction provide detailed information on root biomass and architecture, but are destructive, labour-intensive, and difficult to scale (Maeght et al., 2013; Alani and Lantini, 2020; Wasson et al., 2020). In contrast, emerging technologies, including MR systems, GPR, ERT, and isotope tracing, enable more dynamic, *in situ* observations of root systems (Alani and Lantini, 2020), but require technical expertise, substantial investment, and calibration through ground-truthing. A central conclusion of this perspective is that no single method is sufficient to characterise root systems across space and time. Instead, integrated approaches that combine destructive and non-destructive techniques, observational and analytical tools, and multiple spatial and temporal scales provide the most robust way forward. For example, pairing remote sensing with targeted root sampling improves spatial extrapolation, while combining soil coring with microbial and isotopic analyses strengthens understanding of root contribution to C and nutrient cycling. Despite increasing interest in roots as drivers of pasture performance and ecosystem function, methodological limitations remain a key barrier to their broader incorporation into pastoral research and modelling frameworks.

This perspective highlights that advancing root research requires not only methodological innovation, but also conceptual integration. By framing root phenology as a dynamic functional trait that links plant responses to ecosystem processes, this perspective paper provides a foundation for integrating belowground dynamics into pasture science. Within this framework, root phenology governs the timing and magnitude of resource uptake, C allocation, and soil interactions, thereby influencing system-level outcomes such as productivity, nutrient retention, and resilience. Progress will depend on the integration of root measurements with soil, plant, and microbial data to enable system-level understanding of pasture function. Linking root dynamics with aboveground growth, soil health indicators, and environmental data will support the development of predictive models and decision-support tools tailored to NZ pastoral systems. Embedding root processes within these frameworks is essential for improving representation of belowground dynamics in models and for linking farm-scale management to environmental outcomes.

Looking forward, strategic investment in national capability, long-term datasets, and scalable technologies will be essential. Research that explicitly connects root traits and phenological dynamics to measurable outcomes, including pasture recovery following grazing, nutrient losses, and soil C accumulation, will be essential for informing sustainable intensification of the pastoral sector. Equally important is ensuring that scientific advances translate into practical outcomes through farmer engagement, usability of tools, and alignment with policy and regulatory frameworks. In conclusion, as the role of roots in delivering agroecosystem services becomes increasingly recognised, it is clear that their importance extends beyond biological function to economic and environmental performance. Developing a robust, flexible, and integrated framework for root monitoring and interpretation will be fundamental to meeting the future challenges and opportunities of NZ pasture-based systems.

## Data Availability

The original contributions presented in the study are included in the article/supplementary material. Further inquiries can be directed to the corresponding author.
